# Geometric phases and coherent states for commensurate bichromatic polarization and astigmatic cavities

**DOI:** 10.1515/nanoph-2025-0347

**Published:** 2025-09-22

**Authors:** Miguel A. Alonso

**Affiliations:** 131923 Aix Marseille Univ, CNRS, Centrale Med, Institut Fresnel, UMR 7249, 13397, Marseille, Cedex 20, France; The Institute of Optics, University of Rochester, Rochester, NY, 14627, USA

**Keywords:** geometric phase, bichromatic polarization, coherent states, anisotropic harmonic oscillator

## Abstract

Both the polarization state of coherent bichromatic fields produced by harmonic generation and a class of anisotropic paraxial optical cavities are examples of commensurate two-dimensional harmonic oscillators. The geometric phase for these systems is studied here, both in the classical/ray and quantum/wave regimes. The quantum geometric phase is described in terms of the coherent states of the system, for which recursive expressions are derived that yield the exact result and are numerically stable even for high modal orders.

## 1 Introduction

A commensurate anisotropic harmonic oscillator is a system consisting of two or more degrees of freedom, where each is described by harmonic oscillations of different but commensurate frequencies. There are at least two physical situations in paraxial optics that correspond to commensurate two-dimensional anisotropic harmonic oscillators. The first is that of laser cavities with specific amounts of astigmatism [1], or equivalently to gradient index waveguides with appropriate anisotropic refractive index profiles. Here, the “classical” description corresponds to the behavior of rays under propagation, while the analogues of the quantum eigenstates are the transverse profiles of the wave modes. It has been shown that these cavities accept modes that take the shape of Lissajous curves [1], [2], [3], [4], [5], and that specific cases accept modes whose shapes happen to be separable in parabolic coordinates [6], [7]. These systems have been implemented experimentally by introducing appropriate anisotropic focusing elements in a laser cavity [3], [4], [7]. The second optical realization is not in the spatial field profile but in its polarization state, and corresponds to coherent bichromatic fields with commensurate frequencies [8], [9], [10], [11], obtainable for example through nonlinear harmonic generation [12], [13], [14], [15]. Such fields have been shown theoretically and experimentally to exhibit many polarization distribution patterns and singularities [8], [9], [10], [11], [16]. The classical trajectories are then simply the paths traced by the electric field vector as a function of time, while the quantum eigenstates correspond to exotic states of quantum polarization. Of course, these two types of realization are completely different physically, as are the experimental techniques needed to implement and measure them, and the type of information a measurement can obtain. Nevertheless, their shared underlying model reveals analogous topological features, potentially leading to cross-fertilization of theoretical and experimental ideas.

Commensurate two-dimensional anisotropic harmonic oscillators are a simple yet rich mechanical model that accepts closed-form solutions both in the classical and quantum regimes due to their factorizable nature. They are an example of what has been termed “accidental degeneracy” [17], [18], coming from different combinations between the eigenvalues of the factorizable parts that provide the same sum. They are described by the Hamiltonian

(1) $$\hat{H}=-\frac{1}{2k^{2}}\left(\partial_{x}^{2}+\partial_{y}^{2}\right)+V\left(x,y\right),$$

(2) $$V\left(x,y\right)=V_{2}\frac{m_{x}^{2}x^{2}+m_{y}^{2}y^{2}}{2},$$

where **x** = (*x*, *y*) are two spatial variables, the constants *m*_*x*_ and *m*_*y*_ are nonnegative integers that do not share any prime factors, and *k* = 1/*ℏ* (or 2*π* divided by the wavelength in paraxial optics) is a constant that could be set to unity by using appropriate units but that we retain for asymptotic bookkeeping. Henceforth we choose units for which the mass and the potential coefficient *V*_2_ equal unity.

In this work we study how the concept of geometric (or Pancharatnam-Berry) phase [19] extends to these systems, both in the classical (or ray) and quantum (or wave) domains, and whether this phase corresponds to an area or solid angle over the manifold that describe the different Lissajous oscillation shapes. We also provide a method for calculating exactly and robustly the coherent states of the system associated with each classical oscillation shape. These coherent states serve precisely as the basis for the definition of the quantum geometric phase, which for anisotropic oscillators is shown to be approximately proportional to the classical one only in the limit of highly excited states.

## 2 Classical description

We consider first the classical solutions, which correspond to closed orbits of Lissajous shape for which the position vector **Q** = (*Q*_*x*_, *Q*_*y*_) is composed of two sinusoidals of different frequencies. These orbits can be parametrized as

(3) $$\begin{array}{ll} Q & =\left[\frac{Q_{0}}{m_{x}}\cos\frac{\theta }{2}\cos\left(m_{x}t+\frac{\phi }{2m_{y}}\right),\right.\\ & \;\left.\frac{Q_{0}}{m_{y}}\sin\frac{\theta }{2}\cos\left(m_{y}t-\frac{\phi }{2m_{x}}\right)\right], \end{array}$$

where *Q*_0_ is a constant, *θ* determines the relative amplitude of the oscillations in both directions, and *ϕ* determines the relative phase delay. Note that the orbits are fully covered in a period in *t* of 2*π*. In this respect, varying *ϕ* over an interval of size 2*π* is sufficient to cover the full range of orbit shapes for the given constant *θ*. The momentum **P** = (*P*_*x*_, *P*_*y*_) = *∂*_*t*_ **Q** is given by

(4) $$\begin{array}{ll} P & =-\left[Q_{0}\cos\frac{\theta }{2}\sin\left(m_{x}t+\frac{\phi }{2m_{y}}\right),\right.\\ & \;\left.\;\;\;\;Q_{0}\sin\frac{\theta }{2}\sin\left(m_{y}t-\frac{\phi }{2m_{x}}\right)\right]. \end{array}$$

There are several independent combinations of **Q** and **P** that are constants of the motion, and correspond to generalizations of the Stokes parameters for monochromatic polarization. The first corresponds to the oscillator’s energy:

(5) $$T_{0}=m_{x}^{2}\frac{Q_{x}^{2}}{2}+m_{y}^{2}\frac{Q_{y}^{2}}{2}+\frac{P_{x}^{2}}{2}+\frac{P_{y}^{2}}{2}=\frac{Q_{0}^{2}}{2}.$$

A second invariant is a measure of the mismatch in oscillation amplitudes in both directions/frequencies:

(6) $$T_{1}=m_{x}^{2}\frac{Q_{x}^{2}}{2}-m_{y}^{2}\frac{Q_{y}^{2}}{2}+\frac{P_{x}^{2}}{2}-\frac{P_{y}^{2}}{2}=\frac{Q_{0}^{2}}{2}\cos\theta .$$

The remaining two invariants are not quadratic forms, given the different frequencies of oscillation in the two directions. They can be constructed as the real (*T*_2_) and imaginary (*T*_3_) parts of the combination

(7) $$\begin{array}{ll} T_{2}+i\;T_{3} & \propto {\left(m_{x}Q_{x}-iP_{x}\right)}^{m_{y}}{\left(m_{y}Q_{y}+iP_{y}\right)}^{m_{x}}\\ & =Q_{0}^{m_{x}+m_{y}}\cos^{m_{y}}\frac{\theta }{2}\sin^{m_{x}}\frac{\theta }{2}\exp\left(i\phi \right). \end{array}$$

We can use appropriate powers of *T*_0_ to normalize the remaining parameters *T*_*n*_ according to

(8) $$\begin{array}{ll} \vec{\tau } & =\left(\tau_{1},\tau_{2},\tau_{3}\right)\\ & =\left(\cos\theta ,C\cos^{m_{y}}\frac{\theta }{2}\sin^{m_{x}}\frac{\theta }{2}\cos\phi ,C\cos^{m_{y}}\frac{\theta }{2}\sin^{m_{x}}\frac{\theta }{2}\sin\phi \right), \end{array}$$

where the constant *C* can be chosen, say, so that the maximal value for ${\left(\tau_{2}^{2}+\tau_{3}^{3}\right)}^{1/2}$ is unity:

(9) $$C=\sqrt{\frac{{\left(m_{x}+m_{y}\right)}^{m_{x}+m_{y}}}{m_{x}^{m_{x}}m_{y}^{m_{y}}}}.$$

**Figure 1:**
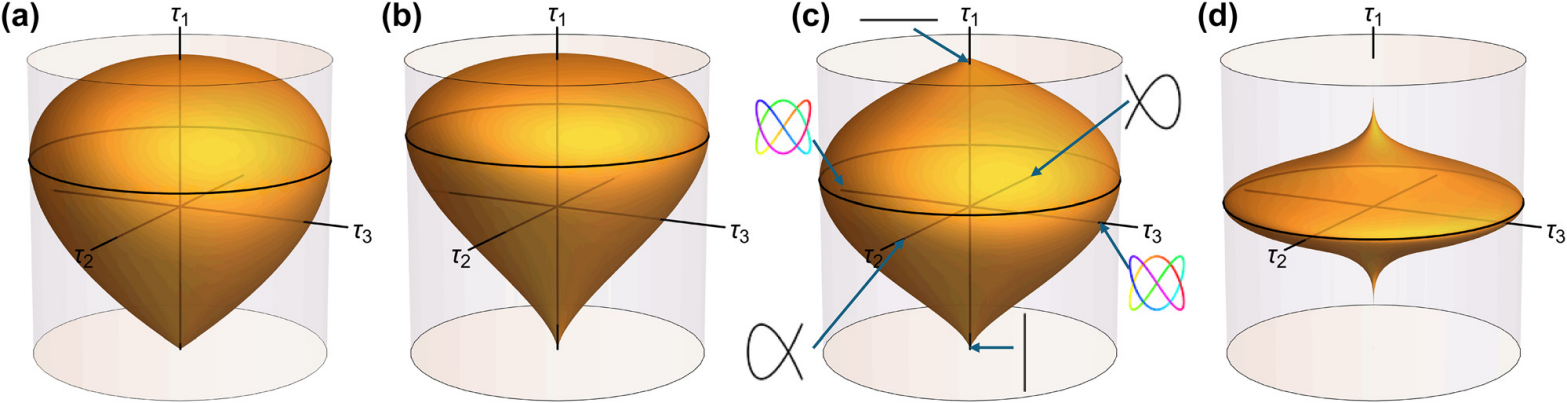
Kummer shapes (orange) for anisotropic oscillators with (a) *m*_*x*_ = 1, *m*_*y*_ = 2, (b) *m*_*x*_ = 1, *m*_*y*_ = 3, (c) *m*_*x*_ = 2, *m*_*y*_ = 3, (d) *m*_*x*_ = 21, *m*_*y*_ = 23. The equator of these shapes is shown as a black circle whose radius is unity, so the shapes can be inscribed in a cylinder of unit radius and height two. In (c) some of the orbits are shown, corresponding to the intersections of the axes with the Kummer shape. Note also that the pole $\vec{\tau }=\left(1,0,0\right)$ is smooth only for *m*_*x*_ = 1, and correspondingly the pole $\vec{\tau }=\left(-1,0,0\right)$ is smooth only for *m*_*y*_ = 1. In fact, the case *m*_*x*_ = *m*_*y*_ = 1 (not shown) corresponds to the standard Poincaré sphere.

The vector $\vec{\tau }$ then defines a manifold known as a Kummer shape [20], which in the case *m*_*x*_ = *m*_*y*_ = 1 reduces to the Poincaré sphere, with the invariants *T*_*n*_ then becoming the Stokes parameters. Each point over this manifold corresponds to an orbit shape, namely a Lissajous curve with a given sense. Figure 1 shows some examples of Kummer shapes, as well as some of the orbits for one of them. By construction, Kummer shapes have rotational symmetry around the *τ*_1_ axis, and one can see that they are widest at *τ*_1_ = *ϵ*, where

(10) $$\epsilon =\frac{m_{y}-m_{x}}{m_{x}+m_{y}}.$$

We refer to this circular cross-section as their equator. Note that (except for the degenerate case *m*_*x*_ = *m*_*y*_ corresponding to the Poincaré sphere) the only geodesics for the Kummer shapes that are contained in planes are precisely this equator as well as the curves of constant *ϕ* (denoted as meridians).

It is worth stressing that only for the isotropic case (*m*_*x*_ = *m*_*y*_ = 1) does the invariant *T*_3_ represent an angular momentum (which is orbital when we are describing cavity modes and spin when we are considering polarization). In fact, if we define the mean angular momentum of the orbit as $L={\left(2\pi \right)}^{-1}\int_{0}^{2\pi }\left(Q_{x}P_{y}-Q_{y}P_{x}\right)\;dt$ , one can easily show that this quantity vanishes exactly except in the isotropic case, for which it gives $L=\left(Q_{0}^{2}/2\right)\sin\theta \sin\phi$ . For anisotropic oscillators, while *T*_3_ does not correspond to an angular momentum, it does share some of its qualitative features: the orbit’s shape depends on the magnitude of *T*_3_ and not on its sign, but this sign determines the sense of circulation. The subset of orbits for which *T*_3_ = 0 (such as those shown in Figure 1c as black curves) are those for which the orbit oscillates back and forth along the same path, so they are the analogues of linear polarizations for the isotropic case (although they are only strictly linear when *T*_2_ also vanishes). If we were studying polarization textures over an extended field, these *T*_3_ = 0 orbits would serve to define the analogues of L-lines or L-surfaces. Note, however, that much of the published work on polarization singularities for bichromatic fields [8], [9], [10], [11] associates to each frequency not a linear but a circular polarization.

## 3 Jones vector and classical geometric phase

We can write the position vector as **Q** = *Q*_0_ Re{diag[exp(−i*m*_*x*_ *t*), exp(−i*m*_*y*_ *t*)]**v**}, where the normalized complex vector **v** (analogous to the Jones vector for optical polarization when *m*_*x*_ = *m*_*y*_ = 1) is defined as

(11) $$v\left(\theta ,\phi \right)=\left[\cos\frac{\theta }{2}\;\exp\left(-i\frac{\phi }{2m_{y}}\right),\;\sin\frac{\theta }{2}\;\exp\;\left(i\frac{\phi }{2m_{x}}\right)\right].$$

In analogy with the isotropic case, we can define a geometric phase associated with transformations of **v**. Let us consider continuous changes corresponding to a parametrization of *θ* and *ϕ* in terms of a continuous variable *η*. The rate of accumulation of geometric phase is then defined as the phase difference between two infinitesimally separated states of polarization:

(12) $$d\Phi_{\text{C}}=\arg\left\{v^{*}\left[\theta \left(\eta \right),\phi \left(\eta \right)\right]\cdot v\left[\theta \left(\eta +d\eta \right)\right.,\phi \left(\eta +d\eta \right)\right\}.$$

By substituting the expression for **v** and simplifying, one gets

(13) $$\begin{array}{ll} d\Phi_{\text{C}} & =\left[-\frac{1}{2m_{y}}\cos^{2}\frac{\theta }{2}+\frac{1}{2m_{x}}\sin^{2}\frac{\theta }{2}\right]\;\partial_{\eta }\phi \;d\eta \\ & =-\frac{m_{x}+m_{y}}{4m_{x}m_{y}}\left(\cos\theta -\epsilon \right)\;\partial_{\eta }\phi \;d\eta . \end{array}$$

**Figure 2:**
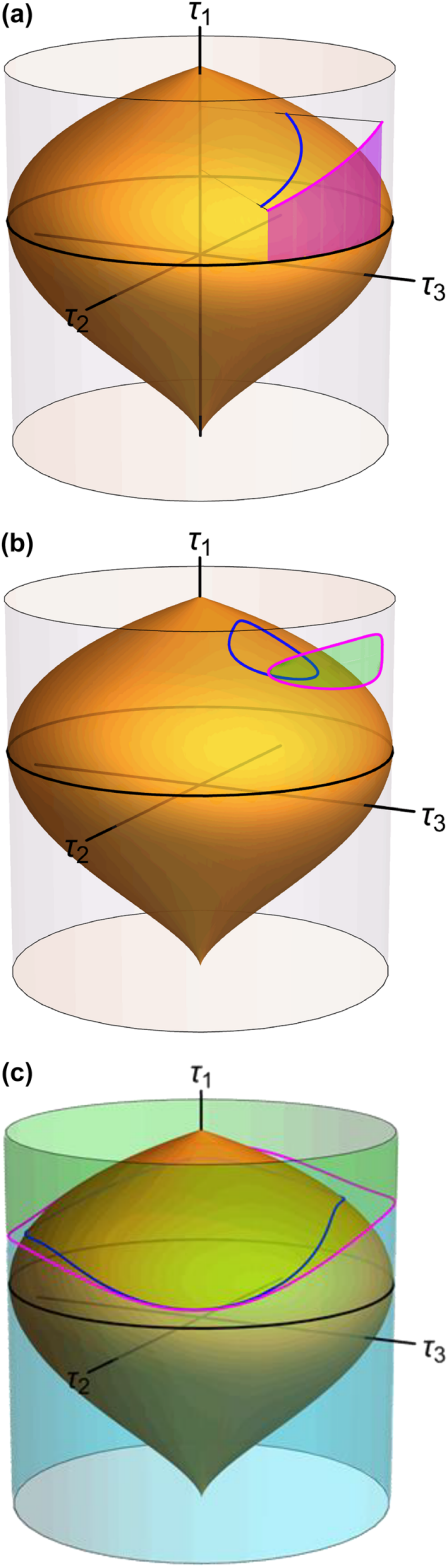
A geometric interpretation for the classical geometric phase. (a) The integral in *η* of (*τ*_1_ − *ϵ*)*∂*_*η*_ *ϕ*d*η* over a given interval corresponds to the area (magenta) accumulated between the equator and the projection of the curve from the Kummer shape onto a cylinder of unit radius centered at the *τ*_1_ axis. (b) For a closed loop that does not surround the *τ*_1_ axis, Ω corresponds to the area (green) enclosed by the projection of the loop onto the cylinder, where the sign is determined by the handedness of the loop. (c) For a closed loop that surrounds the *τ*_1_ axis, Ω_+_ and Ω_−_ correspond to the cylinder’s area above (green) and below (pale blue) the projection of the loop onto the cylinder.

Note that the factor (cos *θ* − *ϵ*) *∂*_*η*_ *ϕ* d*η* can also be written as (*τ*_1_ − *ϵ*) d*ϕ*, suggesting the following geometric interpretation [shown in Figure 2a]: Consider the projection from the Kummer shape onto a cylinder of unit radius centered at the *τ*_1_ axis. The accumulation of geometric phase is then proportional to the area enclosed between the projection of the equator and the projection of the curve traced by *τ*_1_. In the degenerate case *m*_*x*_ = *m*_*y*_ = 1, this area over a cylinder equals the area over the sphere, namely a solid angle, yielding the standard geometric phase for optical polarization. In the general non-degenerate case, on the other hand, the geometric phase does not correspond to an area or solid angle over the Kummer shape.

Note that the rate of accumulation of geometric phase described above is associated with the specific definition of the Jones vector in Eq. (11); different definitions would result from multiplying this one by an arbitrary phase factor that depends on *θ* and *ϕ*. However, when we consider the total phase accumulated over a closed loop, the geometric phase becomes independent of the specific definition and is of a more fundamental nature. Let us consider only loops over the Kummer shape that do not cross themselves. There are two types of such loops:

i) Loops that do not surround the *τ*_1_ axis [illustrated in Figure 2b], for which the initial and final values of *ϕ* coincide. For these, the total geometric phase is
(14) $$\Phi_{\text{C}}=\frac{m_{x}+m_{y}}{4m_{x}m_{y}}\;\Omega ,$$
  where Ω is the area enclosed over the cylinder (or equivalently, over the unit sphere with spherical angles *θ*, *ϕ*), with a sign determined by the handedness of the loop (positive for a right-handed loop in the outward sense).
ii) Loops that surround the *τ*_1_ axis [illustrated in Figure 2c], where the initial and final values of *ϕ* differ by 2*π*. Let us first assume that *ϕ* increases by 2*π* as we complete the loop. The area between the equator and the loop equals 2*π*(1 − *ϵ*) minus the area above the curve, which can be regarded as the enclosed area Ω_+_. Therefore,
(15a) $$\begin{array}{ll} {\bar{\Phi }}_{\text{C}} & =\frac{m_{x}+m_{y}}{4m_{x}m_{y}}\;\left[\Omega_{+}-2\pi \left(1-\epsilon \right)\right]\\ & =\frac{m_{x}+m_{y}}{4m_{x}m_{y}}\;\Omega_{+}-\frac{\pi }{m_{y}}. \end{array}$$
  If, on the other hand, *ϕ* decreases by 2*π* as the loop is closed (i.e., the sense is reversed) one gets
(15b) $${\bar{\Phi }}_{\text{C}}=\frac{m_{x}+m_{y}}{4m_{x}m_{y}}\;\Omega_{-}-\frac{\pi }{m_{x}},$$
  where Ω_−_ corresponds to the cylinder’s area below the curve. Notice that the extra contributions in Eqs. (15) occur even in the isotropic case, where they reflect the nonperiodicity of the Jones vector, namely **v**(*θ*, *ϕ* + 2*π*) ≠ **v**(*θ*, *ϕ*). That is, there is a part of this accumulated phase that is not an intrinsic geometric phase proportional to an enclosed area but a consequence of the parametrization (the bar in ${\bar{\Phi }}_{\text{C}}$ indicating this). For the isotropic oscillator *m*_*x*_ = *m*_*y*_ = 1, v*(*θ*, *ϕ*) ⋅**v**(*θ*, *ϕ* ± 2*π*) = −1 for any *θ* and *ϕ*, and this extra phase of −*π* corresponds exactly to the extra contribution in Eqs. (15), so that the intrinsic part of the geometric phase is still given by Eq. (14). In the anisotropic case, this nonperiodicity is more complex, since v*(*θ*, *ϕ*) ⋅**v**(*θ*, *ϕ* + 2*π*) in general has a magnitude different from unity, and its phase depends on *θ*. These phases take simple forms at the poles, and these forms coincide exactly with the extra terms in Eqs. (15): arg[v*(0, *ϕ*) ⋅**v**(0, *ϕ* + 2*π*)] = −*π*/*m*_*y*_ and arg[v*(*π*, *ϕ*) ⋅**v**(*π*, *ϕ* − 2*π*)] = −*π*/*m*_*x*_.

## 4 Semiclassical wave state construction

We now consider eigenstates for this system in the quantum/wave regime. Asymptotic estimates to these eigenstates can be constructed based on the Lissajous orbits. In order to do so, we must consider two-parameter families of these trajectories, given that the system is two-dimensional. First, we introduce a parameter *ξ* to denote a temporal shift in the orbit according to *t* → *t* + *ξ*. Since we are constructing modes whose shape is invariant in *t*, it is sufficient to consider *t* = 0, so effectively we just replace the physical variable *t* with the delay parameter *ξ*. The second parameter, called here *η*, is used to parametrize both *θ* and *ϕ*. The construction will also require the classical action *S*, which is linked to the positions and momenta by the following equations:

(16) $$\partial_{\xi }S=P\cdot \partial_{\xi }Q,\;\;\;\;\;\;\;\;\partial_{\eta }S=P\cdot \partial_{\eta }Q.$$

In the particular context of classical paraxial optics, where *ξ*, *η* identify a ray, *S* represents the optical path length from a normal to the rays, and Eqs. (16) guarantee that the family of rays constitutes a normal congruence, that is, that the rays are locally perpendicular to a surface element. As shown in Appendix A, these equations have a solution of the form

(17) $$S\left(\xi ,\eta \right)=\Sigma \left(\xi ,\theta ,\phi \right)+S_{\text{GP}}\left(\eta \right),$$

where Σ(*ξ*, *θ*, *ϕ*) is given by a fairly simple closed-form expression in which the only dependence on *η* is within the parameters *θ*, *ϕ*, and *S*_GP_ is related to the geometric phase:

(18) $$S_{\text{GP}}\left(\eta \right)=\frac{Q_{0}^{2}}{4m_{x}m_{y}}\overset{\eta }{\int }\cos\theta \;\partial_{\eta }\phi \;d\eta .$$

We now seek to construct the wave eigenstates based on the orbits. To do this, we use what is known as a semiclassical method, which is a procedure to construct asymptotic estimates of wave fields or quantum wavefunctions by using only the information provided by rays or classical trajectories. While several approaches exist [21], we use one that consists on dressing each orbit with a Gaussian contribution [22], [23] and that does not suffer from singularities at caustics. This approach has been applied to a variety of self-similar Gaussian beams [24], [25], [26], [27] (which correspond to the modes of the isotropic 2D harmonic oscillator), for which it is able to actually reconstruct exact wave fields from the ray description. It is shown in what follows that this exact ray-based wave (or classical-based quantum) construction extends to the anisotropic case, so the results found are not simply asymptotic estimates but true modes. The wave field is expressed as a superposition of Gaussians centered at the ray coordinates **Q**, each presenting a linear phase ramp proportional to the ray momentum **P**, that is,

(19) $$\begin{array}{ll} \Psi \left(x\right) & \propto \iint \sqrt{\frac{\partial \left(\Gamma Q+iP\right)}{\partial \left(\xi ,\eta \right)}}\exp\left\{-k\frac{\left(x-Q\right)\Gamma \left(x-Q\right)}{2}\right.\\ & \;\left.+ik\left[S+\left(x-Q\right)\cdot P\right]\right\}\;d\xi d\eta , \end{array}$$

where Γ is a constant matrix that determines the extent of the Gaussian contributions, whose eigenvalues have positive real parts.

The semiclassical estimate in Eq. (19) incorporates naturally Maslov phase shifts in the phase of the square root of the Jacobian if care is taken when evaluating the square root so that the resulting complex function is continuous. For sufficiently large *k* (namely, for eigenstates of sufficiently high order) the estimate is asymptotically insensitive to the choice of Γ . However, it turns out that analytic results for parts of the integral can be obtained in this case by using the diagonal matrix $\Gamma =\mathrm{diag}\left(m_{x},m_{y}\right)$ , for which exp(−kxΓx/2) corresponds to the ground state of the system. The determinant then reduces to a product of a function of *η* and an exponential in *ξ*:

(20) $$\begin{array}{ll} \frac{\partial \left(\Gamma Q+iP\right)}{\partial \left(\xi ,\eta \right)} & =D\exp\left[i\left(\frac{1}{m_{y}}-\frac{1}{m_{x}}\right)\frac{\phi }{2}\right]\\ & \;\times \exp\left[-i\left(m_{x}+m_{y}\right)\xi \right], \end{array}$$

where

(21) $$D=\frac{Q_{0}^{2}}{4}\;\left\{2\sin\theta \;\partial_{\eta }\phi -i\;\left[m_{x}+m_{y}+\left(m_{x}-m_{y}\right)\cos\theta \right]\;\partial_{\eta }\theta \right\}.$$

The solution to each of the two integrals in Eq. (19) is considered separately in the next two sections.

## 5 Coherent states

Given that both D and *S* have closed form expressions for their dependence on *ξ*, the integral in *ξ* for the semiclassical estimate can be solved first. Let us then rewrite Eq. (19) as

(22) $$\Psi \left(x\right)\propto \int \sqrt{D}\;\exp\left(i\Theta_{\text{GP}}\right)\;U_{n}\left(x,\theta ,\phi \right)\;d\eta ,$$

where Θ_GP_(*η*) = *kS*_GP_ + (1/*m*_*y*_ − 1/*m*_*x*_)*ϕ*/4, and $U_{n}\left(x,\theta ,\phi \right)$ is the wavefunction contribution associated with the orbit specified by *θ* and *ϕ*, given by the following integral in *ξ* over an interval of length 2*π*:

(23) $$\begin{array}{ll} U_{n}\left(x,\theta ,\phi \right) & =\int \exp\left\{-k\frac{\left(x-Q\right)\Gamma \left(x-Q\right)}{2}-\frac{m_{x}+m_{y}}{2}i\xi \right.\\ & \;\left.+ik\left[\Sigma +\left(x-Q\right)\cdot P\right]\right\}\;d\xi . \end{array}$$

It is shown in Appendix B that the periodicity of the integrand imposes the quantization condition

(24) $$Q_{0}=\sqrt{\frac{2n+m_{x}+m_{y}}{k}},$$

where *n* is a non-negative integer. With this condition, the integral can be calculated in closed form, as shown in Appendix B, giving a result expressible in terms of the components of the Jones vector **v**:

(25) $$\begin{array}{ll} U_{n}\left(x,\theta ,\phi \right) & =-2\pi \exp\;\left[-\frac{k}{2}\left(m_{x}x^{2}+m_{y}y^{2}\right)\right.\\ & \;\left.-\frac{kQ_{0}^{2}}{4}\left(\frac{|v_{x}{|}^{2}}{m_{x}}+\frac{|v_{y}{|}^{2}}{m_{y}}\right)\right]\\ & \;\times F_{n}\left(kQ_{0}v_{x}x,kQ_{0}v_{y}y,\right.\\ & \;\;\left.-\frac{kQ_{0}^{2}v_{x}^{2}}{4m_{x}},-\frac{kQ_{0}^{2}v_{y}^{2}}{4m_{y}}\right), \end{array}$$

where *F*_*n*_(*α*_*x*_, *α*_*y*_, *β*_*x*_, *β*_*y*_) is an *n*th-order four-variable polynomial composed of terms proportional to $\alpha_{x}^{\mu_{x}}\alpha_{y}^{\mu_{y}}\beta_{x}^{\nu_{x}}\beta_{y}^{\nu_{y}}$ , for all combinations of non-negative integer powers for which *m*_*x*_(*μ*_*x*_ + 2*ν*_*x*_) + *m*_*y*_(*μ*_*y*_ + 2*ν*_*y*_) = *n*. This polynomial satisfies the recurrence relations (for *ρ* = *x*, *y*):

(26) $$\partial_{\alpha_{\rho }}F_{n}=F_{n-m_{\rho }},\;\;\;\;\partial_{\beta_{\rho }}F_{n}=F_{n-2m_{\rho }},$$

(27) $$F_{n}=\underset{\rho =x,y}{\sum }m_{\rho }\frac{\alpha_{\rho }F_{n-m_{\rho }}+2\beta_{\rho }F_{n-2m_{\rho }}}{n},$$

with *F*_*n*__<__0_ = 0 and *F*_0_ = 1. The relation in Eq. (27) is particularly convenient for the fast and numerically robust computation of these polynomials when considering high orders [28], the computation time being proportional to *n*.

The solutions $U_{n}$ just found are not simply asymptotic estimates but exact eigenstates of the Hamiltonian. This is shown by using the recurrence relations to find the constant eigenvalue:

(28) $$\begin{array}{ll} \frac{\hat{H}\;U_{n}}{U_{n}} & =\frac{\left[-k^{-2}\left(\partial_{x}^{2}+\partial_{y}^{2}\right)+m_{x}^{2}x^{2}+m_{y}^{2}y^{2}\right]U_{n}}{2\;U_{n}}\\ & =\frac{m_{x}+m_{y}}{2k}+\underset{\rho =x,y}{\sum }m_{\rho }\frac{\alpha_{\rho }\partial_{\alpha_{\rho }}F_{n}+2\beta_{\rho }\partial_{\alpha_{\rho }}^{2}F_{n}}{kF_{n}}\\ & =\frac{m_{x}+m_{y}}{2k}+\underset{\rho =x,y}{\sum }m_{\rho }\frac{\alpha_{\rho }F_{n-m_{\rho }}+2\beta_{\rho }F_{n-2m_{\rho }}}{kF_{n}}\\ & =\frac{2n+m_{x}+m_{y}}{2k}. \end{array}$$

**Figure 3:**
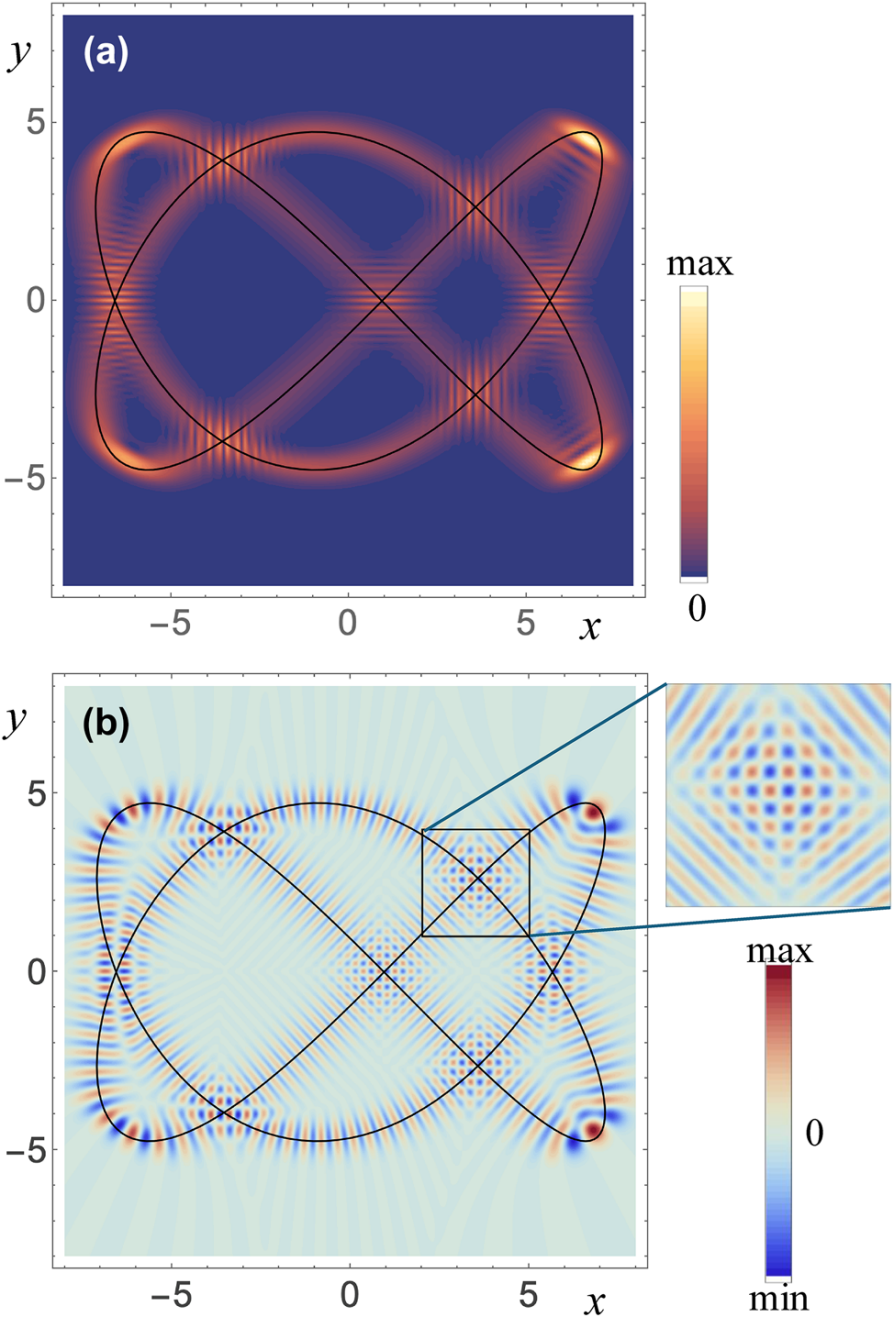
Coherent modes associated with a Lissajous orbit. (a) Modulus squared and (b) real part of a coherent mode with *n* = 200, *m*_*x*_ = 2, *m*_*y*_ = 3, *θ* = *π*/2 and *ϕ* = 3*π*/8. The classical orbit is overlaid on both parts as a black curve. In (b), the inset shows a detail of the mode’s structure near a crossing of the orbit with itself.

This result holds for any *θ* and *ϕ*, so all states with equal *n* are degenerate and can be combined to construct other eigenstates with the same eigenvalue. The states $U_{n}$ can be regarded as coherent states, and their wave profile mimics the Lissajous shape of the orbit for the corresponding values of *θ* and *ϕ*, as illustrated in Figure 3. Some of these states have been produced and measured experimentally as cavity modes [3], [4]. Expressions for these coherent states have been given as specific combinations involving Wigner d coefficients [2], [29], [30], [31]. The different approach taken here where the orbit is dressed with appropriate Gaussians gives an expression in terms of a polynomial that can be computed directly in a numerically robust way by using the recurrence relation in Eq. (27).

The recurrence relations given above show that, following the ground state *n* = 0, the first nonzero eigenstate corresponds to *n* = min(*m*_*x*_, *m*_*y*_), and eigenstates exist only for values of *n* that are nonnegative integer combinations of *m*_*x*_ and *m*_*y*_. Incidentally, note that for the isotropic case *m*_*x*_ = *m*_*y*_ (= 1), this recurrence relation reduces to one with two (rather than four) terms on the right-hand side, and can be transformed into that for Hermite polynomials. This is not surprising since the coherent states for the isotropic oscillator are expressible in terms of Hermite polynomials of a complex expression involving the inner product of the position and Jones vectors [24], [32]. In the context of bichromatic polarization, *n* is related to the number of photons in the state, but it is not necessarily equal to it: the state is composed of all possible numbers of photons *i*_*x*_ + *i*_*y*_ for which *i*_*x*_ *m*_*x*_ + *i*_*y*_ *m*_*y*_ = *n*. In fact, for *θ* ≠ 0, *π*, the coherent states $U_{n}$ are in general entangled in the two frequency components (i.e., non-separable in *x* and *y*), except in special cases where *n* is only composed as an integer multiple of one of the frequencies *m*_*ρ*_ without involving the other.

Finally, note that the recurrence relations in Eqs. (26) and (27) can be combined to arrive at the following relation for the coherent states themselves:

(29) $$2nU_{n}=\underset{\rho =x,y}{\sum }Q_{0}v_{\rho }\left(km_{\rho }\rho -\partial_{\rho }\right)U_{n-m_{\rho }}.$$

The operators acting on the coherent states on the right hand side are clearly creation operators on each of the Cartesian variables, which cause an increase in the total index *n* not of unity but of *m*_*x*_ or *m*_*y*_. We can then see that, to get to a given order *n*, we cannot simply apply the same operator repeatedly, but we have to consider all the combinations of the operators for each of the cartesian variables/frequencies that cause the state to arrive at that order.

## 6 Semiclassical quantization of geometric phase

The calculation of a generic eigenstate in Eq. (22) involves a weighted superposition of coherent states. Of course, any such superposition is an eigenstate, but this semiclassical formula is constructed such that neighboring coherent states are superposed asymptotically in phase along the caustics, so that the resulting waveform mimics the classic structure. If we consider a closed integral such that the integrand is a periodic function of *η*, this requirement of constructive interference of contiguous coherent states implies the accumulation of a phase that must be quantized to guarantee phase self-consistency when closing the integral.

We again consider the closed (non-self-crossing) path over the Kummer shape traced by *θ*(*η*), *ϕ*(*η*). As discussed in Section 3, we consider separately the two types of loop:

i) Loops that do not surround the *τ*_1_ axis, for which the initial and final values of *ϕ* coincide. Notice that D accumulates a phase of ± 2*π* upon closing the loop (the sign depending on the loop’s sense), and hence $\sqrt{D}$ accumulates a phase of ± *π*. The periodicity of the integrand then requires that Θ_GP_ and therefore *kS*_GP_ accumulate an odd multiple of *π*, so that the area Ω enclosed by the loop projected to the cylinder is discretized according to
(30) $$\Omega =-\int \cos\theta \;\partial_{\eta }\phi \;d\eta =\frac{4m_{x}m_{y}\pi }{2n+m_{x}+m_{y}}\left(2\mu +1\right),$$
  for integer *μ*.

ii) Loops that surround the *τ*_1_ axis, where the initial and final values of *ϕ* differ by 2*π*. In this case, D does not accumulate a phase. However, an increase of 2*π* in *ϕ* causes $U_{n}$ to accumulate a phase $\Phi_{U}$ , shown in Appendix B to be given by
(31) $$\Phi_{U}=\frac{i_{y}m_{y}-i_{x}m_{x}}{m_{x}m_{y}}\pi$$
  with *i*_*x*_ and *i*_*y*_ being non-negative integers such that *i*_*x*_ *m*_*x*_ + *i*_*y*_ *m*_*y*_ = *n*. Note that the choice of *i*_*x*_ and *i*_*y*_ is typically not unique: if *n* is sufficiently large, one can change *i*_*x*_ → *i*_*x*_ − *νm*_*y*_, *i*_*y*_ → *i*_*y*_ + *νm*_*x*_ for any integer *ν* that allows keeping these two numbers non-negative; however, this change simply leads to $\Phi_{U}\to \Phi_{U}+2\pi \nu$ , so the specific choice of the integers *i*_*x*_ and *i*_*y*_ (within their allowed values) is inconsequential. The total phase accumulated by the integrand in Eq. (22), given by
(32) $$\begin{array}{ll} \Theta_{\text{GP}}+\Phi_{U} & =\frac{2n+m_{x}+m_{y}}{4m_{x}m_{y}}\int \cos\theta \;\partial_{\eta }\phi \;d\eta \\ & \;+\left(\frac{1}{m_{y}}-\frac{1}{m_{x}}\right)\frac{\pi }{2}+\Phi_{U}, \end{array}$$
  must be an integer multiple of 2*π*. Let us denote by Ω_+_ the cylinder area above the loop:
(33) $$\Omega_{+}=\int \left(1-\cos\theta \right)\;\partial_{\eta }\phi \;d\eta .$$
  This area is then quantized as
(34a) $$\Omega_{+}=\frac{4m_{x}m_{y}\pi }{2n+m_{x}+m_{y}}\left(2\mu +\frac{2i_{y}}{m_{x}}+\frac{1}{m_{y}}\right),$$
  where in order to arrive to the last step we used the relation *n* = *i*_*x*_ *m*_*x*_ + *i*_*y*_ *m*_*y*_. We can, without loss of generality, choose the smallest possible allowed value of *i*_*y*_. Note that, had we chosen instead to use the complementary area below the curve Ω_−_ = 4*π* − Ω_+_ we would get a similar result but with the subindices *x* and *y* swapped:
(34b) $$\Omega_{-}=\frac{4m_{x}m_{y}\pi }{2n+m_{x}+m_{y}}\left(2\mu +\frac{2i_{x}}{m_{y}}+\frac{1}{m_{x}}\right).$$

The quantization conditions for the areas enclosed by the curves in the two cases, given, respectively, in Eqs. (30) and (34), resemble each other except for the offsets inside the parentheses. Notice that Eq. (34a) or Eq. (34b) can only fully coincide with Eq. (30) if *m*_*y*_ or *m*_*x*_ equal unity, respectively, and *n* is an integer multiple of both *m*_*x*_ and *m*_*y*_ (since then *i*_*y*_ or *i*_*x*_ can be chosen to be zero). This suggests that, for cases where *n* is an integer multiple of *m*_*x*_ *m*_*y*_, the difference in the quantization condition has a topological origin and is related to whether the Kummer shape’s pole associated with the area under consideration [namely, $\vec{\tau }=\left(\pm 1,0,0\right)$ for Ω_±_] is smooth or not; if it is smooth, the quantization is not different from the one for a loop not surrounding the *τ*_1_ axis.

## 7 Coherent state overlap, norm, and Husimi representation

We now calculate the overlap between any two coherent states $\left\langle U_{n}^{\left(1\right)}|U_{n}^{\left(2\right)}\right\rangle$ , where the superindices indicate different parameters *θ*_1_, *ϕ*_1_ and *θ*_2_, *ϕ*_2_ for each. By using the definition of the polynomials *F*_*n*_ in terms of derivatives of an exponential in Eq. (57), the overlap integrals can be solved in closed form using standard Gaussian formulas:

(35) $$\begin{array}{ll} \left\langle U_{n}^{\left(1\right)}|U_{n}^{\left(2\right)}\right\rangle & =\iint U_{n}{\left(x,\theta_{1},\phi_{1}\right)}^{*}U_{n}\left(x.\theta_{2},\phi_{2}\right)\;dx\;dy\\ & =\frac{4\pi^{3}}{k\sqrt{m_{x}m_{y}}}\exp\left(-\frac{2n+m_{x}+m_{y}}{4}\right.\\ & \;\left.\times \underset{\rho =x,y}{\sum }\frac{|v_{1\rho }{|}^{2}+|v_{2\rho }{|}^{2}}{m_{\rho }}\right)\\ & \;\times G_{n}\left(\frac{2n+m_{x}+m_{y}}{2}v_{1x}^{*}v_{2x},\right.\\ & \;\;\left.\frac{2n+m_{x}+m_{y}}{2}v_{1y}^{*}v_{2y}\right), \end{array}$$

where

(36) $$G_{n}\left(w_{x},w_{y}\right)={\left.\left\{\frac{\partial_{u_{1}}^{n}\partial_{u_{2}}^{n}}{{\left(n!\right)}^{2}}\exp\left[\underset{\rho =x,y}{\sum }\frac{{\left(u_{1}u_{2}\right)}^{m_{\rho }}w_{\rho }}{m_{\rho }}\right]\right\}\right|}_{u_{1}=u_{2}=0},$$

is a polynomial with two arguments, which can be shown to satisfy the recurrence formula

(37) $$\begin{array}{ll} G_{n} & =\frac{1}{n^{2}}\left(\underset{\rho =x,y}{\sum }m_{\rho }w_{\rho }G_{n-m_{\rho }}\right.\\ & \;\left.+\underset{\rho_{1}=x,y}{\sum }\underset{\rho_{2}=x,y}{\sum }w_{\rho_{1}}w_{\rho_{2}}G_{n-m_{\rho_{1}}-m_{\rho_{2}}}\right), \end{array}$$

with *G*_*n*<0_ = 0 and *G*_0_ = 1. Like *F*_*n*_, this polynomial can be computed rapidly and in a numerically stable form using this type of recurrence relation.

The overlap formula in Eq. (35) allows calculating the norm of the coherent states $U_{n}$ . By evaluating this equation for $U_{n}^{\left(1\right)}=U_{n}^{\left(2\right)}=U_{n}$ (corresponding to the angles *θ*, *ϕ*), one can verify that the result is independent of *ϕ*, but that in general it depends on *θ* (except in the isotropic case *m*_*x*_ = *m*_*y*_ = 1). In fact, the norm of the coherent states tends to zero in the limit *θ* → 0(*π*) unless *n* is an integer multiple of *m*_*x*_(*m*_*y*_). This is easy to understand, since in this limit the oscillator involves a single frequency, and *n* must be an integer multiple of this frequency for the solution to exist.

**Figure 4:**
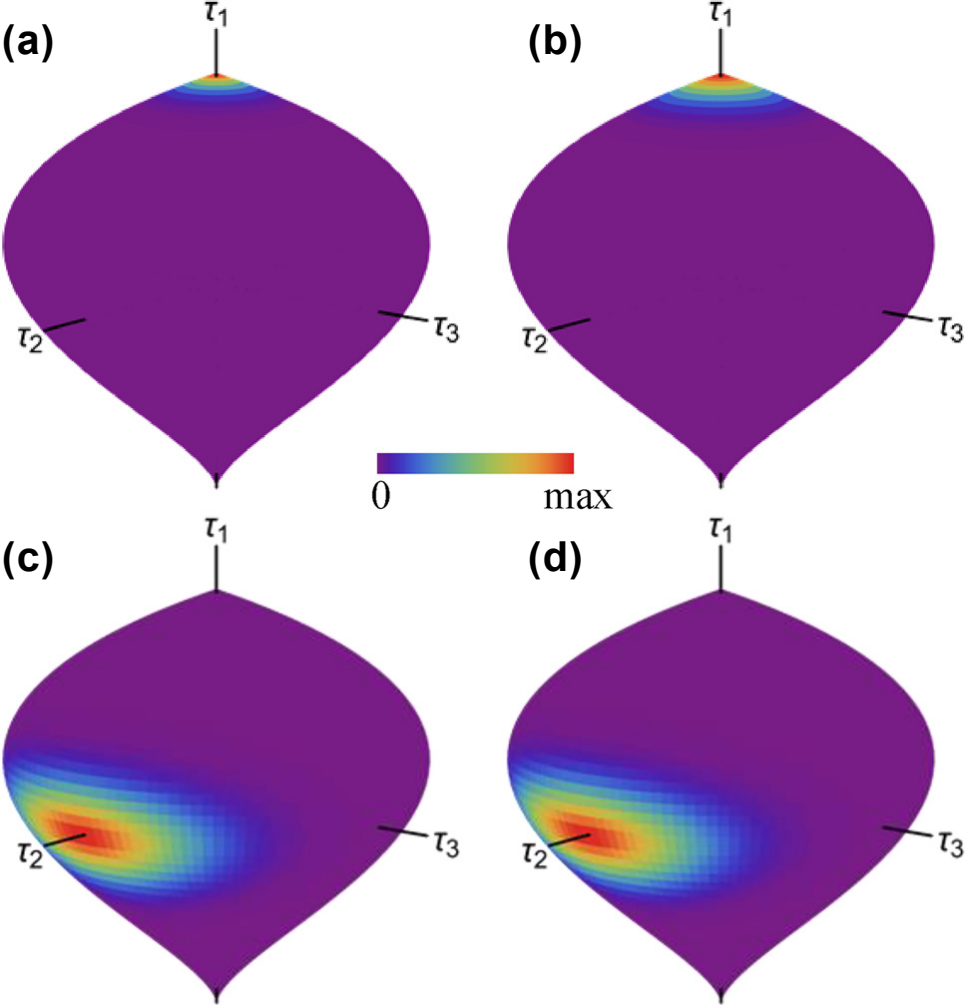
Husimi representation for *m*_*x*_ = 2, *m*_*y*_ = 3 for coherent states with *ϕ* = 0, for (a,b) *θ* = 10^−5^ ≈ 0 and (c,d) *θ* = *π*/2, and where (a,c) *n* = 204 (an integer multiple of *m*_*x*_ and *m*_*y*_), and (b,d) *n* = 205 (not an integer multiple of *m*_*x*_ or *m*_*y*_).

The coherent states $U_{n}$ can be used to construct a Husimi distribution representation of arbitrary modes Ψ with the same *n* according to

(38) $$H\left(\theta ,\phi \right)=\frac{|〈U_{n}\left(\theta ,\phi \right)|\Psi 〉{|}^{2}}{〈U_{n}\left(\theta ,\phi \right)|U_{n}\left(\theta ,\phi \right)〉},$$

similar to that used for the isotropic oscillator, for characterizing quantum states of polarization [33] or structured Gaussian beams [34]. This distribution can be represented over the surface of the corresponding Kummer shape. Figure 4 shows this distribution for the case *m*_*x*_ = 2, *m*_*y*_ = 3 for a wavefunction Ψ proportional to a coherent state $U_{n}$ with *ϕ* = 0 and for both *θ* ≈ 0 (a,b) and *θ* = *π*/2 (c,d). In all cases, the maximum is located around the point that represents the corresponding orbit. Note, however, that the coherent state *θ* = 0 is not allowed except when *n* is an integer multiple of *m*_*x*_, which is the case in (a,c) corresponding to *n* = 204, but not in (b,d) corresponding to *n* = 205. For this reason, a state was used in (a,c) with *θ* = 10^−5^ rather than one corresponding exactly to the pole. Similarly, when evaluating Eq. (38) when *n* is not an integer multiple of *m*_*x*_(*m*_*y*_), the Husimi distribution cannot be evaluated exactly at *θ* = 0(*π*). This irregular behavior at the pole makes the maximum of the Husimi function appreciably more extended in (b) than in (a), despite *n* being marginally larger for the latter. In contrast, the maxima in (c) and (d) are essentially identical (the latter being in theory very slightly more localized due to the larger value of *n*).

When applied to more complex modes, the Husimi distributions become more extended and can present zeros at a number of points. In the symmetric case (*m*_*x*_ = *m*_*y*_ = 1 the location of these zeros is sufficient to characterize the state (up to a global factor, of course) and is known as the Majorana constellation [33], [34]. The number of zeros in the commensurate anisotropic case is *N* if *n* = *Nm*_*x*_ *m*_*y*_.

## 8 Quantum geometric phase

We now consider the rate of accumulation of a “quantum” geometric phase by the coherent states as their parameters trace a closed trajectory in *θ* and *ϕ*. This geometric phase, defined as

(39) $$\Phi_{\text{Q}}=\oint \arg\left\{\left\langle U_{n}\left[\theta \left(\eta \right),\phi \left(\eta \right)\right]|U_{n}\left[\theta \left(\eta +d\eta \right),\phi \left(\eta +d\eta \right)\right]\right\rangle \right\},$$

can be calculated using the overlap expression in Eq. (35). Note that the factors multiplying *G*_*n*_ in Eq. (35) are real and positive, so they do not contribute to the phase. We start by considering the phase accumulation due to infinitesimal changes in *θ* and *ϕ* given by d*θ* = *∂*_*η*_ *θ*d*η* and d*ϕ* = *∂*_*η*_ *ϕ*d*η*, respectively. We then evaluate the polynomials at $w_{\rho }=\left(kQ_{0}^{2}/2\right)v_{\rho }^{*}{|}_{\theta ,\phi }v_{\rho }{|}_{\theta +d\theta ,\phi +d\phi }$ , i.e.,

(40a) $$w_{x}=\frac{2n+m_{x}+m_{y}}{2}\cos^{2}\frac{\theta }{2}\left(1-i\frac{d\phi }{2m_{y}}\right),$$

(40b) $$w_{y}=\frac{2n+m_{x}+m_{y}}{2}\sin^{2}\frac{\theta }{2}\left(1+i\frac{d\phi }{2m_{x}}\right).$$

That is, like for the classical geometric phase, only differentials in *ϕ* play a role, so it suffices to compare the rate of phase accumulation in *ϕ* as a function of *θ*. Recall that, for the classical geometric phase, this rate equals −(*m*_*x*_ + *m*_*y*_)/(4*m*_*x*_ *m*_*y*_)(*τ*_1_ − *ϵ*).

It is useful to consider first the special case of the isotropic oscillator, corresponding to *m*_*x*_ = *m*_*y*_ = 1. One can see that the recurrence relation in Eq. (37) becomes then a two-term relation given by $G_{n}=\left(WG_{n-1}+W^{2}G_{n-2}\right)/n^{2}$ with *W* = *w*_*x*_ + *w*_*y*_ = (*n* + 1) exp(−i*τ*_1_ d*ϕ*/2) for infinitesimal changes, following Eqs. (8). This recursion relation has a solution of the form *G*_*n*_ ∝ *W* ^*n*^, so the rate of phase accumulation in *ϕ* by *G*_*n*_ is exactly −*n*/2 cos *θ*, and hence the exact quantum geometric phase is simply *n* times the classical one: Φ_Q_ = *n*Φ_C_.

For anisotropic oscillators, on the other hand, the rate of phase accumulation takes a more complicated form that typically requires numerical calculation. There are, however, limiting situations where this rate can be calculated analytically: at the poles, one of the two expressions in Eqs. (8) vanishes, reducing the recurrence relation in Eq. (37) to $G_{n}=\left(m_{\rho }w_{\rho }G_{n-m_{\rho }}+w_{\rho }^{2}G_{n-2m_{\rho }}\right)/n^{2}$ , where *ρ* = *x* for *θ* = 0 and *ρ* = *y* for *θ* = *π*. If *n* is an integer multiple of *m*_*ρ*_, we find $G_{n}\propto w_{\rho }^{n/m_{\rho }}$ , giving a rate of phase accumulation of ∓ *n*/(2*m*_*x*_ *m*_*y*_), the sign corresponding to *θ* being 0 or *π*. This suggests the following asymptotic relation for the rate of quantum geometric phase accumulation with *ϕ*:

(41) $$\partial_{\phi }\Phi_{\text{Q}}\approx -\frac{n}{2m_{x}m_{y}}\;\tau_{1}.$$

That is, ignoring the offset of *ϵ* in the classical geometric phase (which is inconsequential for loops not surrounding the *τ*_1_ axis), the quantum geometric phase is approximately (*m*_*x*_ + *m*_*y*_)*n*/2 times larger than the classical one for sufficiently large *n*. (Note that this approximate result indeed reduces to Φ_Q_ = *n*Φ_C_ for the isotropic case *m*_*x*_ = *m*_*y*_ = 1.)

**Figure 5:**
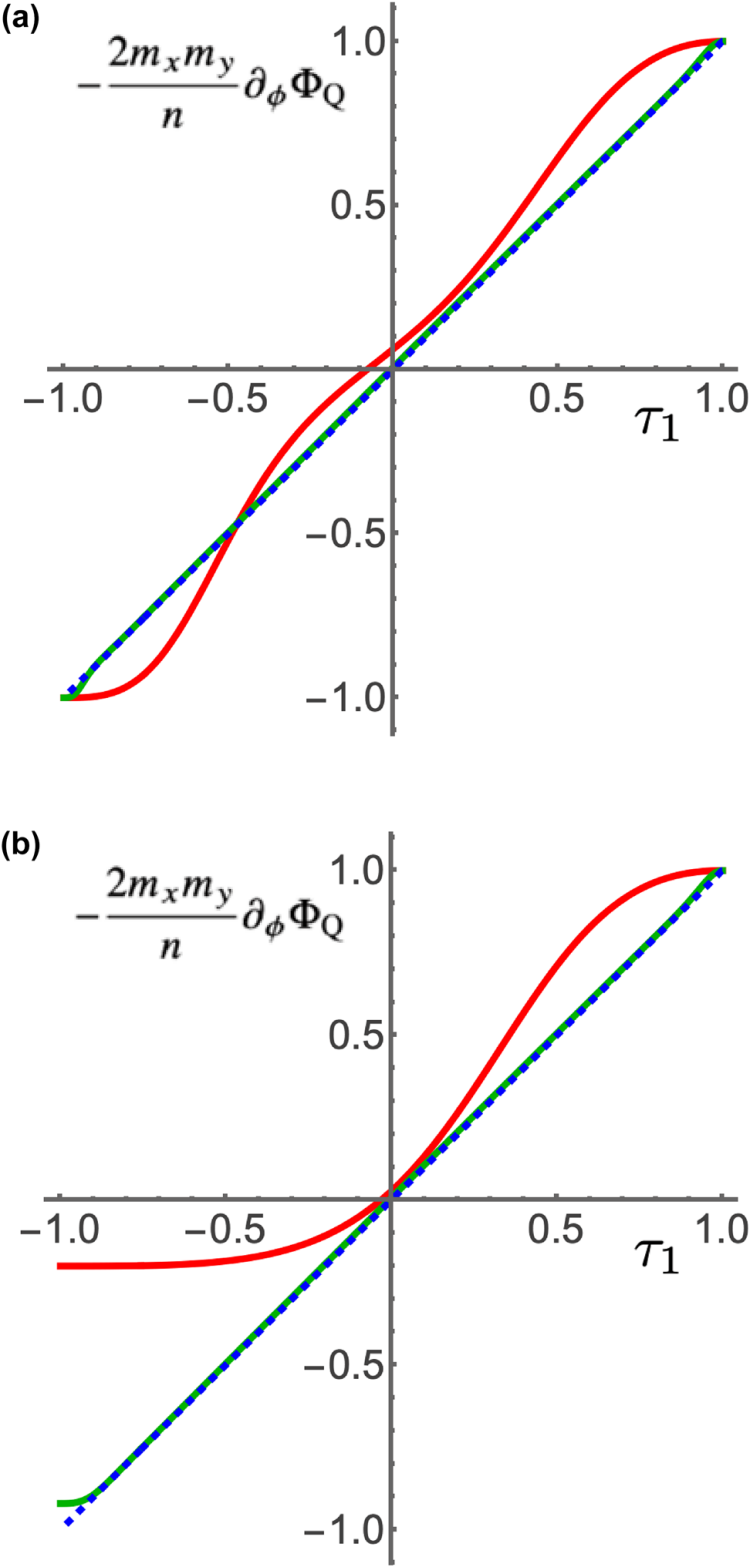
Plots of −2*m*_*x*_ *m*_*y*_ *∂*_*ϕ*_Φ_Q_/*n* versus *τ*_1_ for *m*_*x*_ = 2, *m*_*y*_ = 3. The values of *n* are (a) 12 (red), 102 (green) and 1002 (dashed blue), and (b) 10 (red), 100 (green) and 1000 (dashed blue).

Figure 5 shows numerically calculated plots of −2*m*_*x*_ *m*_*y*_ *∂*_*ϕ*_Φ_Q_/*n* versus *τ*_1_, for *m*_*x*_ = 2, *m*_*y*_ = 3, and for increasing values of *n* that are (a) integer multiples of *m*_*x*_ and *m*_*y*_, and (b) integer multiples of *m*_*x*_ but not of *m*_*y*_. In both cases, the approximate result in Eq. (41) is validated for sufficiently large *n*, but in the latter case there is a noticeable departure from linearity within a region around *τ*_1_ = −1. This discrepancy can be understood by the fact that the relation $G_{n}\propto w_{\rho }^{n/m_{\rho }}$ mentioned earlier for *θ* = 0, *π* is actually not valid if *n*/*m*_*ρ*_ is not an integer. That is, no quantized state exists that involves only one frequency *m*_*ρ*_ if *n* is not an integer multiple of that frequency. To obtain the behavior in the close vicinity of this forbidden value, we must consider *θ* being close to its endpoints but not equal to them, so that one of the two expressions in Eqs. (8) is much smaller than the other but not zero. The dominant contribution to *G*_*n*_ will then be approximately proportional to $w_{\rho }^{i_{\rho }}w_{\bar{\rho }}^{i_{\bar{\rho }}}$ , with *ρ* = *x*, *y*, $\bar{\rho }=y,x$ and where the nonnegative integers *i*_*ρ*_ and $i_{\bar{\rho }}$ satisfy the relation $i_{\rho }m_{\rho }+i_{\bar{\rho }}m_{\bar{\rho }}=n$ with $i_{\bar{\rho }}$ being as small as possible. Therefore, the correct result near |*τ*_1_| = 1 is −2*m*_*x*_ *m*_*y*_ *∂*_*ϕ*_Φ_Q_/*n* ≈ *m*_*x*_ *i*_*x*_ − *myi*_*y*_, for *i*_*x*_ as small as possible if *τ*_1_ ≈ 1 and *i*_*y*_ as small as possible if *τ*_1_ ≈ − 1. This explains why in Figure 5b, for which *n* is not an integer multiple of *m*_*y*_, the curves start at (2*m*_*x*_ − 2*m*_*y*_)/*n* = −0.2 for *n* = 10, (2*m*_*x*_ − 32*m*_*y*_)/*n* = −0.92 for *n* = 100, and (2*m*_*x*_ − 332*m*_*y*_)/*n* = −0.992 for *n* = 1000.

## 9 Geometric phase for the comparison of states

So far we have consider the accumulation of geometric phase due to continuous transformations of the orbit shapes. For the isotropic oscillator, these continuous transformations are easily realizable: one can consider, for example, the passage of polarized light through birefringent media with arbitrary eigenpolarizations as a way to transform continuously the polarization state of a monochromatic beam. For commensurate anisotropic oscillators, on the other hand, the situation is different. Uniform shifts in *ϕ* (corresponding to rotations around *τ*_1_, which is the only axis of rotational symmetry of the Kummer shape) are easy to implement, through birefringence for bichromatic polarization or by detuning the frequency ratio for the cavity. On the other hand, operations that change *θ* are not easy to envisage, especially if we want these operations to be unitary. It can therefore be challenging to implement experiments to observe the accumulation of geometric phase described so far.

There is, however, a simple manifestation of geometric phase that is not based on continuous transformations but on the comparison of the phases of at least three states. Suppose that we have three different states, labelled as A, B, and C. We start by measuring the interference between A and B, and we adjust the global phase of B so that it is “in phase” with A, meaning that the constructive interference of A and B is maximized. We then do the same between B and C, giving C the necessary phase to maximize its interference with B. Despite having optimized the phase agreement between A and B and between B and C, in general the phase agreement between C and A is not optimal, in that an extra phase would be needed to make their interference as constructive as possible. This difference is due to the geometric phase. For the isotropic oscillator (e.g. for standard monochromatic polarization), this phase is given by half the area over the sphere enclosed by the geodesic triangle defined by the three states. We now consider the general anisotropic case.

Let us start by considering the classical geometric phase, defined as

(42) $$\Phi_{\text{C}}=\arg\left[\left(v_{\text{A}}^{*}\cdot v_{\text{B}}\right)\left(v_{\text{B}}^{*}\cdot v_{\text{C}}\right)\left(v_{\text{C}}^{*}\cdot v_{\text{A}}\right)\right].$$

A geometric interpretation for this phase follows from rewriting the Jones vector in Eq. (11) as

(43) $$\begin{array}{ll} v\left(\theta ,\phi \right) & =\exp\;\left(i\frac{m_{y}-m_{x}}{4m_{x}m_{y}}\phi \right)\\ & \;\times \left[\cos\frac{\theta }{2}\;\exp\;\left(-i\frac{m_{y}+m_{x}}{4m_{x}m_{y}}\phi \right),\right.\\ & \;\;\left.\sin\frac{\theta }{2}\;\exp\;\left(i\frac{m_{y}+m_{x}}{4m_{x}m_{y}}\phi \right)\right]. \end{array}$$

Note that the global phase factor at the front has no effect on Φ_C_, since it cancels for each state in Eq. (42). The second, vectorial part is mathematically identical to a standard Jones vector for a single frequency, except that *ϕ* is scaled by a rational factor. Therefore, a geometric interpretation for Φ_C_ corresponds to half the area enclosed inside the geodesic triangle (or polygon, for more than three states) over a sphere (or more precisely, a spherical section) whose azimuthal angle is not *ϕ* but $\frac{m_{y}+m_{x}}{4m_{x}m_{y}}\phi$ . Note, however, that geodesics over this sphere do not correspond to geodesics over the projected cylinder nor over the Kummer shape. In particular, for a given set of states corresponding to three sets of angles *θ* and *ϕ*, the classical geometric phase does not change if we exchange the values of *m*_*x*_ and *m*_*y*_.

We now consider the quantum geometric phase, given by

(44) $$\Phi_{\text{Q}}=\arg\left[\left\langle U_{n}^{\left(A\right)}|U_{n}^{\left(B\right)}\right\rangle \left\langle U_{n}^{\left(B\right)}|U_{n}^{\left(C\right)}\right\rangle \left\langle U_{n}^{\left(C\right)}|U_{n}^{\left(A\right)}\right\rangle \right].$$

In general, this phase has no simple closed form expression or geometric interpretation, but it can be easily calculated by using Eq. (35). Like for the continuous case, for *n* ≫ *m*_*x*_, *m*_*y*_ we expect the asymptotic relation

(45) $$\Phi_{\text{Q}}\approx \frac{m_{x}+m_{y}}{2}n\Phi_{\text{C}}.$$

**Figure 6:**
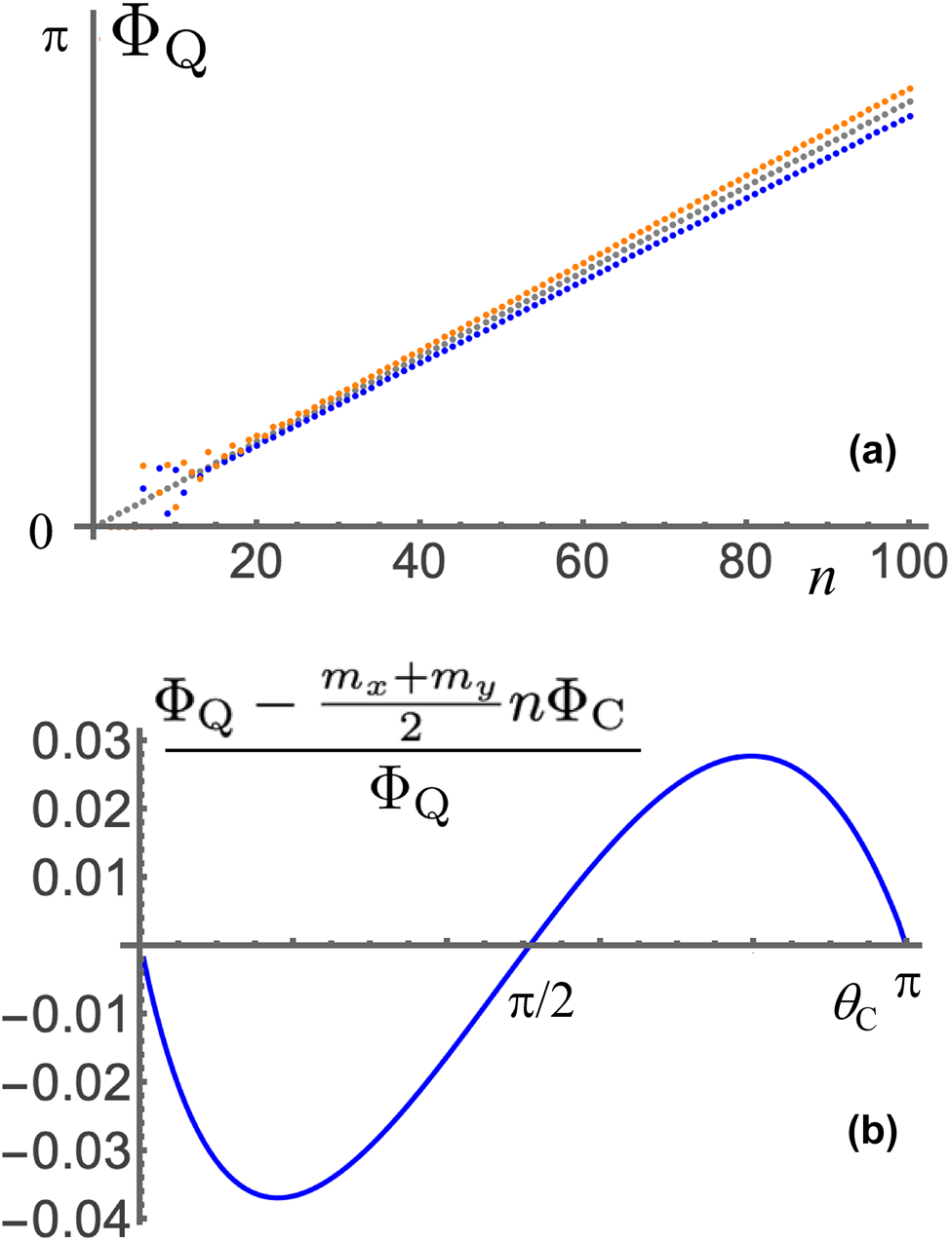
Quantum geometric phases between three states. (a) Quantum geometric phase between the states with angles (*θ*, *ϕ*) given by (*π*/2, 0), (*π*/2, *π*/4), and (*π*/4, 0), as a function of *n* and for *m*_*x*_ = 2, *m*_*y*_ = 3 (blue), and *m*_*x*_ = 3, *m*_*y*_ = 2 (orange). The gray line corresponds to the corresponding estimate in Eq. (44). (b) Relative error between the quantum geometric phase and the asymptotic estimate for *n* = 1000, *m*_*x*_ = 2, *m*_*y*_ = 3, and for the same states as in (a) except that the third state is replaced by (*θ*_C_, 0) with *θ*_C_ varying from 0 to *π*.

Figure 6a considers the geometric phases for all *n* up to 100 corresponding to three states with (*θ*, *ϕ*) given by (*π*/2, 0), (*π*/2, *π*/4), and (*π*/4, 0), for two anisotropic oscillators with *m*_*x*_ = 2, *m*_*y*_ = 3 and *m*_*x*_ = 3, *m*_*y*_ = 2. We observe that, for small *n* the quantum geometric phases differ significantly from the asymptotic estimate and from each other, but later they grow approximately linearly in *n*, mimicking Eq. (44) but with a slope that is slightly higher or lower depending on whether *m*_*x*_ or *m*_*y*_ is larger, somewhat reflecting the Kummer shape’s asymmetry in contrast to the classical geometric phase. To understand this asymmetry, Figure 6b shows the difference between Φ_Q_ and the estimate in Eq. (44), normalized by Φ_Q_ for the states with (*θ*, *ϕ*) given by (*π*/2, 0), (*π*/2, *π*/4), and (*θ*_C_, 0) as a function of *θ*_C_ for *n* = 1000. The maximum error in slope is of about 3–4 %, but for *n* = 1000 this means a phase difference of about *π*/2.

## 10 Concluding remarks

The semiclassical treatment provided here for the two-dimensional commensurate anisotropic harmonic oscillator leads to two main results. The first is a robust exact construction of the coherent states associated with the orbits, in which each “particle” is dressed by an appropriate Gaussian wave packet. Remarkably, the integral can be solved in closed form and the resulting polynomials satisfy simple recurrence relations that facilitate their computation. These coherent states can be used for constructing more complicated modes, as well as for defining Husimi representations and Majorana constellations. Appendix C provides a simple implementation for Wolfram’s Mathematica of this recurrence relation, as well as for the one that gives access to the overlap between two coherent states.

The second result is the definition of both classical and quantum geometric phases for these systems. In the semiclassical limit (corresponding to highly excited states, for which *n* ≫ *m*_*x*_, *m*_*y*_), the quantum geometric phase is proportional to the classical one, the proportionality constant being (*m*_*x*_ + *m*_*y*_)*n*/2. Like in the case of the isotropic oscillator, these phases are proportional to enclosed areas, but these do not correspond to the area of the curved manifold that naturally represents the constants of the motion for the system (the Kummer shape). When we are considering continuous transformations, the area can be associated with that of a projection from the *τ*_1_ axis onto a cylinder of unit radius. (For the isotropic oscillator this projected area happens to equal that of the sphere.) When we consider instead the phase between a discrete set of states, the geometric interpretation requires considering geodesic polygons over a sphere where the azimuthal angle is scaled.

These results can also be applied to commensurate oscillators in which the oscillations of each frequency are not along a line but trace a more general elliptic shape. One such example are bichromatic superpositions of counterrotating circular polarizations [3], [8], [9], [10], [16], [35]. A general class of these results can be obtained by applying a linear canonical transformation (or Collins’ ABCD formula) [36], such as an astigmatic fractional Fourier transform whose axes are not aligned with *x* and *y*. This transformation is unitary, so the results for the classical and quantum geometric phases still apply. Further, because these integral transformations involve kernels that are exponentials of imaginary quadratic polynomials, they can be performed in closed form when applied to the expressions in Appendix B.

Finally, note that the semiclassical approach used here can also be applied to anisotropic commensurate harmonic oscillators in three or more dimensions, probably leading also to closed forms for the coherent states and to asymptotic expressions for the geometric phase. It will be interesting to see if in the three-dimensional case this phase accepts a geometric interpretation similar to one proposed recently for isotropic 3D oscillators [37].

## Appendix A: Equations for the action

We can solve Eqs. (16) by first setting *ξ* to a constant such as zero and integrating in *η* the equation for *∂*_*η*_ *S* to find a function *S*_0_(*η*), and then adding to this solution the integration in *ξ* (from zero) of the equation for *∂*_*ξ*_ *S* for fixed *η* (and hence fixed *θ* and *ϕ*). That is, we write $S\left(\xi ,\eta \right)=S_{0}\left(\eta \right)+\bar{S}\left(\xi ,\theta ,\phi \right)$ , where each of the two parts satisfies one of the following equations:

(46)
$$\begin{array}{ll} \partial_{\eta }S_{0} & =\frac{Q_{0}^{2}}{8m_{x}m_{y}}\left[\left(m_{x}\sin\frac{\phi }{m_{x}}+m_{y}\sin\frac{\phi }{m_{y}}\right)\sin\theta \;\partial_{\eta }\theta \right.\\ & \;\left.+2\left(\cos\theta +\sin^{2}\frac{\theta }{2}\cos\frac{\phi }{m_{x}}-\cos^{2}\frac{\theta }{2}\cos\frac{\phi }{m_{y}}\right)\;\partial_{\eta }\phi \right], \end{array}$$

and

(47)
$$\begin{array}{ll} \partial_{\xi }S & =Q_{0}^{2}\left[\cos^{2}\frac{\theta }{2}\sin^{2}\left(m_{x}\xi +\frac{\phi }{2m_{y}}\right)\right.\\ & \;\left.+\sin^{2}\frac{\theta }{2}\sin^{2}\left(m_{y}\xi -\frac{\phi }{2m_{x}}\right)\right]. \end{array}$$

This latter equation can be integrated to give a closed-form expression of the form

(48)
$$\begin{array}{ll} \bar{S}\left(\xi ,\theta ,\phi \right) & =\frac{Q_{0}^{2}}{2}\left[\xi -\frac{\sin\left(m_{x}\xi \right)}{m_{x}}\cos\left(m_{x}\xi +\frac{\phi }{m_{y}}\right)\cos^{2}\frac{\theta }{2}\right.\\ & \;\left.-\frac{\sin\left(m_{y}\xi \right)}{m_{y}}\cos\left(m_{y}\xi -\frac{\phi }{m_{x}}\right)\sin^{2}\frac{\theta }{2}\right]. \end{array}$$

Equation (46) does not have a generic closed-form primitive, but it can be separated into a part that is a total derivative and one proportional to the derivative of the classical geometric phase:

(49)
$$\begin{array}{ll} \partial_{\eta }S_{0} & =-\partial_{\eta }\left\{\frac{Q_{0}^{2}}{4}\left[\frac{Im\left(v_{x}^{2}\right)}{m_{x}}+\frac{Im\left(v_{y}^{2}\right)}{m_{y}}\right]\right\}\\ & \;+\frac{Q_{0}^{2}}{4m_{x}m_{y}}\cos\theta \;\partial_{\eta }\phi . \end{array}$$

These results lead to the expression in Eq. (17), where the integrable part is given by

(50)
$$\Sigma \left(\xi ,\theta ,\phi \right)=\bar{S}\left(\xi ,\theta ,\phi \right)-\frac{Q_{0}^{2}}{4}\left[\frac{Im\left(v_{x}^{2}\right)}{m_{x}}+\frac{Im\left(v_{y}^{2}\right)}{m_{y}}\right].$$

## Appendix B: Derivation of the coherent states and their recurrence relations

The wavefunction contribution $U_{n}\left(x,\theta ,\phi \right)$ defined in Eq. (23) can be rewritten as

(51)
$$\begin{array}{ll} U_{n}\left(x,\theta ,\phi \right) & =\exp\left(-k\frac{m_{x}x^{2}+m_{y}y^{2}}{2}\right)\\ & \;\times \int \exp\left\{kx\cdot \left(\Gamma Q+iP\right)-\frac{m_{x}+m_{y}}{2}i\xi \right.\\ & \;\left.+ik\left[\Sigma -Q\cdot P\right]\right\}\;d\xi , \end{array}$$

the integral being over an interval of length 2*π*.

By substituting *u* = exp(−i*ξ*) (so that d*ξ* = i*u* ^−1^d*u*), the integral can be changed to one along the unit circle over the complex *u* plane:

(52)
$$\begin{array}{ll} U_{n}\left(x,\theta ,\phi \right) & =i\exp\;\left[-\frac{k}{2}\left(m_{x}x^{2}+m_{y}y^{2}\right)-\frac{kQ_{0}^{2}}{4}\left(\frac{|v_{x}{|}^{2}}{m_{x}}+\frac{|v_{y}{|}^{2}}{m_{y}}\right)\right]\\ & \;\times \oint \frac{\exp\left(\alpha_{x}u^{m_{x}}+\alpha_{y}u^{m_{y}}+\beta_{x}u^{2m_{x}}+\beta_{y}u^{2m_{y}}\right)}{u^{n+1}}\;du, \end{array}$$

with **v** = (*v*_*x*_, *v*_*y*_) defined in Eq. (11), and where

(53)
$$n=\frac{kQ_{0}^{2}-\left(m_{x}+m_{y}\right)}{2},$$

(54)
$$\alpha_{x}=kQ_{0}v_{x}\;x,\;\;\;\;\;\;\;\;\;\;\;\;\alpha_{y}=kQ_{0}v_{y}\;y,$$

(55)
$$\beta_{x}=-k\frac{{\left(Q_{0}v_{x}\right)}^{2}}{4m_{x}},\;\;\;\;\beta_{y}=-k\frac{{\left(Q_{0}v_{y}\right)}^{2}}{4m_{y}}.$$

Note that the integral in *u* over a unit circle in the complex plane is uniquely defined only if the integrand is fully periodic, that is, if the power −*n* − 1 of *u* in the first factor is an integer. This is true if the quantization condition in Eq. (24) is satisfied with *n* a nonnegative integer. Further, the remarkable fact that the exponential includes only a few powers of *u*, all of them positive, means that the integral can be evaluated in closed form by using the residue theorem, since the only singularity is a pole of order *n* + 1 at the origin. The result can then be written as

(56)
$$\begin{array}{ll} U_{n}\left(x,\theta ,\phi \right) & =-2\pi \exp\;\left[-\frac{k}{2}\left(m_{x}x^{2}+m_{y}y^{2}\right)\right.\\ & \;\left.-\frac{kQ_{0}^{2}}{4}\left(\frac{|v_{x}{|}^{2}}{m_{x}}+\frac{|v_{y}{|}^{2}}{m_{y}}\right)\right]\\ & \;\times F_{n}\left(kQ_{0}v_{x}x,kQ_{0}v_{y}y,\right.\\ & \;\;\left.-\frac{kQ_{0}^{2}v_{x}^{2}}{4m_{x}},-\frac{kQ_{0}^{2}v_{y}^{2}}{4m_{y}}\right), \end{array}$$

where *F*_*n*_ is an *n*th-order four-variable polynomial defined as

(57)
$$\begin{array}{ll} & F_{n}\left(\alpha_{x},\alpha_{y},\beta_{x},\beta_{y}\right)\\ & \;=\frac{1}{2\pi i}\oint \frac{\exp\left(\alpha_{x}u^{m_{x}}+\alpha_{y}u^{m_{y}}+\beta_{x}u^{2m_{x}}+\beta_{y}u^{2m_{y}}\right)}{u^{n+1}}\;du\\ & \;=\frac{1}{n!}{\left[\partial_{u}^{n}\exp\left(\alpha_{x}u^{m_{x}}+\alpha_{y}u^{m_{y}}+\beta_{x}u^{2m_{x}}+\beta_{y}u^{2m_{y}}\right)\right]}_{u=0}. \end{array}$$

It is easy to show that *F*_*n*_ satisfies the recurrence relations in Eqs. (26) and (27), the proof for the latter consisting on using integration by parts in the integral form in Eq. (57).

It turns out that the coherent state $U_{n}$ is not periodic in *ϕ*, but it accumulates a phase factor if *ϕ* increases by 2*π*:

(58)
$$U_{n}\left(x,\theta ,\phi +2\pi \right)=U_{n}\left(x,\theta ,\phi \right)\exp\left(i\Phi_{U}\right).$$

Note that the factors in front of *F*_*n*_ in the definition of $U_{n}$ are real, but *F*_*n*_ itself is complex. As mentioned earlier, this polynomial is composed of terms proportional to $\alpha_{x}^{\mu_{x}}\alpha_{y}^{\mu_{y}}\beta_{x}^{\nu_{x}}\beta_{y}^{\nu_{y}}$ , for non-negative integer combinations *m*_*x*_(*μ*_*x*_ + 2*ν*_*x*_) + *m*_*y*_(*μ*_*y*_ + 2*ν*_*y*_) = *n*. By using the forms in Eqs. (54) and (55) taken by the arguments of the polynomial, we see that the phase of each term is

(59)
$$\left(\mu_{y}+2\nu_{y}\right)\frac{\phi }{2m_{x}}-\left(\mu_{x}+2\nu_{x}\right)\frac{\phi }{2m_{y}}=\frac{i_{y}m_{y}-i_{x}m_{x}}{2m_{x}m_{y}}\phi ,$$

with *i*_*ρ*_ = *μ*_*ρ*_ + 2*ν*_*ρ*_ a non-negative integer such that *i*_*x*_ *m*_*x*_ + *i*_*y*_ *m*_*y*_ = *n*. From here we deduce that the phase accumulated by the polynomial following an increase of 2*π* in *ϕ* is given by

(60)
$$\Phi_{U}=\frac{i_{y}m_{y}-i_{x}m_{x}}{m_{x}m_{y}}\pi .$$

## Appendix C: Mathematica code for the recurrence relations

We give here simple code for implementing in Wolfram’s Mathematica the recurrence relations for the coherent states and their overlap. The recurrence formula for the coherent state given in Eq. (27) can be implemented as

while that for the overlap of coherent states given in Eq. (37) can be implemented as
